# Cognitive Control in Suicide Ideators and Suicide Attempters

**DOI:** 10.3389/fpsyg.2020.595673

**Published:** 2020-12-23

**Authors:** Silje Støle Brokke, Nils Inge Landrø, Vegard Øksendal Haaland

**Affiliations:** ^1^Clinical Neuroscience Research Group, Department of Psychology, Faculty of Social Sciences, University of Oslo, Oslo, Norway; ^2^Department of Psychiatry, Sørlandet Hospital HF, Kristiansand, Norway; ^3^Division of Psychiatry, Diakonhjemmet Hospital, Oslo, Norway

**Keywords:** cognitive control, suicide ideation, suicide attempt, suicidal behaviour, cognitive rigidity, inhibition, cognitive shift, attention control

## Abstract

There is a need to understand more of the risk factors involved in the process from suicide ideation to suicide attempt. Cognitive control processes may be important factors in assessing vulnerability to suicide. A version of the Stroop procedure, Delis–Kaplan Executive Function System (D-KEFS) Color–Word Interference Test (CWIT) and Behavior Rating Inventory of Executive Function (BRIEF-A) were used in this study to test attention control and cognitive shift, as well as to assess everyday executive function of 98 acute suicidal psychiatric patients. The Columbia Suicide History Form (CSHF) was used to identify a group of suicide ideators and suicide attempters. Results showed that suicide attempters scored lower on attention control than suicide ideators who had no history of attempted suicide. The self-report in the BRIEF-A inventory did not reflect any cognitive differences between suicide ideators and suicide attempters. A logistic regression analysis showed that a poorer attention control score was associated with the suicide attempt group, whereas a poorer cognitive shift score was associated with the suicide ideation group. The results found in this study suggest that suicide attempters may struggle with control of attention or inhibiting competing responses but not with cognitive flexibility.

## Introduction

Suicide is a worldwide growing public health problem (Bertolote and Fleischmann, 2015). It is difficult to explain exactly why some people choose to end their life, but suicide can best be understood as a multicausal act (Shermer, 2018). William’s Cry of Pain model explains suicidal behavior as a response to a stressful situation that creates feelings of defeat and is judged to be both inescapable and having no chance of rescue (Williams, 2014).

Suicidal behavior has different levels of severity. Definitions of the behavior range from deliberate self-harm to completed suicide (Turecki and Brent, 2016). In this study, we refer to suicidal behavior as thoughts of committing suicide, plans to commit suicide, and suicidal attempt (Bruffaerts et al., 2010). However, it can be difficult to assess the intent of self-harm behaviors and an ambivalence toward life or death. To assess suicide risk, clinicians often try to understand the relevance and severity of different risk factors, the severity of suicidal ideation, and the intention to attempt suicide (Bryan and Rudd, 2006).

Suicidal behavior has previously been understood as a symptom of an existing psychiatric diagnosis, such as depression, psychosis, and personality disorder (Rogers et al., 2017). Yet, it can now be argued that understanding suicidal behavior as only a symptom of disorders is too limited to cover all dimensions of suicidal behavior (Rogers et al., 2017). A meta-analysis of psychological autopsy studies comprising 3,275 suicide completers showed that they had different psychiatric diagnoses and 458 suicide completers that did not have a psychiatric disorder (Arsenault-Lapierre et al., 2004). Sisti et al. (2020) argue that the risk for suicidal behavior needs explicit attention. It would be ideal if diagnostic systems like the DSM-5 and ICD-11 included an independent category for suicidal behavior. A codable construct for suicide risk could include both clinical and research based detection of risk factors (Sisti et al., 2020).

There are many known risk factors that contribute toward suicidal behavior. Beyond the deficits that are also associated with depression, other cognitive deficits have been found to be related to suicidal behavior (Dour et al., 2011; McGirr et al., 2012; Jager-Hyman et al., 2014; Neacsiu et al., 2018). Cognitive functions found to be related to suicidal behavior include attention control, long-term memory, and working memory (Keilp et al., 2013). Research that compare depressed patients with and without suicidal ideation suggest that suicidal mental states may result from dysfunctional executive decision making (Marzuk et al., 2005).

Richard-Devantoy et al. (2014) performed a meta-analysis of neuropsychological markers of vulnerability to suicidal behavior in mood disorders. They included 25 studies comprising 2,323 participants and analyzed patient performance on the verbal fluency test, the Iowa gambling test, and the Stroop test. They found that suicide attempters performed lower on the Iowa-gambling test and verbal fluency test in comparison to patient controls and healthy controls. Stroop performance was also lower in suicide attempters compared to patient controls and healthy controls. The researchers concluded that cognitive control processes might be important factors of vulnerability to suicidal behavior.

One study that has looked at different cognitive control related factors in suicidal behavior is by Loyo et al. (2013). They compared suicide attempters with depressive symptoms, depressive participants without suicide attempts, and non-depressed participants. They used the Behavior Rating Inventory of Executive Function (BRIEF-A; Roth et al., 2005) and conducted a correlation analysis of the scores of different clinical measurements. Suicide attempters scored higher than depressed non attempters on cognitive shift, but there was no difference between these two groups on any of the other cognitive index measurements. The study found significant differences between both of the patient groups and the non-depression group on all the cognitive measures of BRIEF. The study also found that suicide attempters needed more time and made more errors in an original Stroop condition in comparison to non-depressed participants, but no difference was found between the patient groups. These results suggest that patients with suicide attempt can struggle with attention control as measured by the Stroop condition and score higher on cognitive shift as measured by BRIEF than non-depressed individuals (Loyo et al., 2013).

Several studies refer to the interference condition in Stroop as inhibition. There is however a debate to the inhibitory process involved in Stroop. The interference effect in the Stroop test could have several meanings. Mac Leod argues that the effect could be that one response slows down the other. Therefore, the effect could be interference or the effect could be a result of inhibition (MacLeod et al., 2003). In this study, the effect of the interference condition is referred to as attention control.

As suicidal behavior is a broad concept, it is important to differentiate between the severity of various suicidal behaviors. There is a difference between thinking about suicide and actually attempting suicide. It is important to understand more of the factors involved in the progression from suicide thought to suicide behavior. Saffer and Klonsky (2018) conducted a comprehensive literature search and found only 14 studies that compared suicide attempters and ideators on neurocognitive abilities. Most studies suggested that attempters and ideators score similarly across tests of neurocognitive abilities, with the exceptions of inhibition and decision making. More specifically, one logistic regression analysis of 40 suicide ideators and 37 ideators with suicide attempts showed that suicide attempters exhibited poorer attention control as measured by the Stroop condition, but better problem solving ability in a neuropsychological battery than suicide ideators (Burton et al., 2011).

Saffer and Klonsky (2018) state the need for research to compare the neurocognitive predictors of ideation and suicide attempts. The study asks for larger samples, as several studies only had around 10 participants or less in each group, and for the use of validated neurocognitive measures. Their study also asks for longitudinal prediction of suicidality outcomes and for utilized cross-sectional design.

The current study aims to contribute to filling in the knowledge gaps in the field of cognitive control and suicidal behavior. The study will seek to identify whether the level of cognitive control is related to suicide ideation and suicide attempt. To accomplish this, patients with suicide ideation and no history of suicide attempt and patients with suicide ideation and a history of suicide attempt will be compared.

We predict that suicide ideators who have made one or more suicide attempts will have lower scores of attention control in both self-reports and standard tests than suicide ideators who have not made any suicide attempts. It is further predicted that suicide attempters will score higher than suicide ideators on cognitive shift.

## Materials and Methods

### Participants

The participants were acute psychiatric patients referred to one of the crisis resolution teams at Sørlandet Hospital in Southern Norway. The crisis resolution team aims to help people experiencing a mental health crisis usually related to suicidal and acute mental illness issues. The inclusion period ran from May 2014 to August 2017. The study was designed as a natural study of suicidality in acute psychiatric patients. The participants included in the analysis for this specific paper were patients with suicide ideation and no suicide attempt, and suicide attempters who all had completed a set of different cognitive tests (*N* = 98). The main inclusion criteria to participate were to be between the ages of 18 and 65 years and to have been referred to psychiatric help for suicide risk. Exclusion criteria were severe substance abuse and the inability to read, speak, or write Norwegian. The study was approved by the Regional Committee for Medical and Health Research Ethics (2013/1664/REK sør øst D).

### Measures

#### Columbia Suicide History Form

The CSHF was selected for this study to assess the severity of an individual’s suicide ideation and suicide attempt. The method has been validated as a suitable assessment of suicidal ideation and suicidal behavior in both clinical and research settings. It has shown good convergent and divergent validity with other multi-informant suicidal ideation and behavior scales (Posner et al., 2011).

The CSHF was developed by Mann and Quendo at the Conte Center for the Neuroscience of Mental Disorders at New York State Psychiatric Institute (Posner et al., 2008). The form is structured as a screening interview with five questions on suicide ideation, seven questions on intensity of ideation, six questions on suicidal behavior, and two questions on lethality evaluations of actual attempts. The interview covers both suicidal behavior during the previous month and lifetime history of suicidal behavior for all the questions. Suicide attempts are also categorized by the first, latest, and most deadly attempt. Regarding the research aim of comparing suicide attempters and suicide ideators, these two groups were defined by the CSHF’s categories for suicide ideation and suicide attempt. The clinicians involved in the research process and data collection were all trained to complete the screening interview. Their training included a video made by the developers of the CSHF in addition to an observation of an interview between a researcher and a patient.

#### Montgomery and Aasberg Depression Rating Scale

The MADRS is a standardized rating scale designed to detect symptoms of an ongoing depression (Montgomery and Åsberg, 1979). The validity of the method has been confirmed in several studies (Leucht et al., 2017). MADRS includes 10 different phenomena related to depression, which clinicians assess with a rating from 0 to 6. This depression scale is the standard rating scale used in the division of mental health at Sørlandet Hospital and appropriate training was given.

#### Color–Word Interference Test

We used the Delis–Kaplan Executive Function System (D-KEFS) CWIT (Delis et al., 2001) as a measure of cognitive control. The test is easy and fast to administer and is valuable for testing cognitive speed. CWIT consists of four conditions named: color naming, word reading, inhibition, and inhibition or switching. The participants are asked to complete each task as quickly as possible without making mistakes. The completion time was measured in seconds and error rates were calculated to the nearest hundredth for the four conditions. The scores were further scaled by the norms in Delis et al. (2001) *Examiner’s Manual*.

#### Behavior Rating Inventory of Executive Function

Behavior Rating Inventory of Executive Function (BRIEF; Roth et al., 2005) is a 75 item self-reported questionnaire used in assessment of executive function. The participants are instructed to consider a statement of behavior and report whether it is often a problem for them, sometimes a problem for them, or never a problem for them. The questionnaire produces a behavioral rating measure specifically designed to assess executive skills in natural, everyday environments. BRIEF-A has been found to be a reliable and valid screening tool for assessing executive function (Ciszewski et al., 2014). The clinical scales of this questionnaire are inhibit, shift, emotional control, self-monitor, initiate, working memory, plan or organize, task monitor, and organization of materials. These nine scales make up a global executive composite (GEC). The sum of the clinical scales (inhibit, shift, emotional control, and self-monitor) form the Behavioral Regulation Index (BRI) and the sum of the scores from the scales (initiate, working memory, plan or organize, task monitor, and organization of materials) comprise the Metacognition Index (MI). The raw scores of the sub-indexes in BRIEF-A were transformed to T-scores using the *BRIEF–A Professional Manual*. A higher score indicates greater degrees of executive dysfunction.

### Procedure

Participants were asked to participate in the study after their first meeting with clinicians at the hospital. They were given a written information form and explained the nature of the study. If interested, they were given a consent form to sign and informed that they had the right to change their mind at any time. The researcher and participant would then find a time and place suitable for each participant to do the interview and tests. Some participants preferred to meet in a consultation room at the ward where they were staying at the time, some preferred the researcher’s office, and some preferred a home visit.

At the hospital, all patients who meet with the crisis resolution team go through a clinical assessment of suicide risk where clinicians follow Norwegian’s national guidelines of suicide prevention for assessment. The Columbia History Form was used to generate categories for suicide ideation and suicide attempt. These categories constituted the main variables of this study.

### Statistical Analysis

Statistical analyses were performed using SPSS for Mac (Version 26). A descriptive analysis was conducted by comparing the mean of the scores from the CWIT and BRIEF, and the control variables (gender, years of education, and depression score) for the two suicide behavior groups. *T*-tests and chi-square tests were used where appropriate. The standard deviation and *p*-value of the different groups of suicide risk were reported. The analysis of demographic and clinical characteristics was conducted for description purposes only, thus no alpha adjustment for multiple comparisons was made. A binary logistic regression analysis was conducted to test the predictability of the cognitive control and control variables for suicide ideators and suicide attempters. The Nagelkerke R Square was used for the binary logistic regression analysis to evaluate the suitability of the models.

## Results

The mean age for participants in the study was 36.3 years, and mean years of education were 13. The mean depression score (MADRS) was 24.6, which indicates a moderate level of depression. The most common diagnostic groups represented were affective disorders, neurotic and stress related disorders, personality disorders, and disorders related to substance use. The most used psychotropics were hypnotics (Table 1).

**Table 1:** Characteristics of patients included in the analysis, *n* = 98.

	*N*	%	Mean	SD
Age			36.3	14.4
**Gender**				
Male	45	46		
Female	53	54		
Education in years			13.0	3.3
Depression score			24.6	7.4
**Main diagnosis, ICD −10**				
F10-19 disorders related to substance use	8	8.2		
F20-29 disorders related to psychosis	1	1		
F30-39 affective disorders	40	41.1		
F40-48 neurotic and stress related disorders	25	25.3		
F50 eating disorder	1	1		
F60-69 personality disorders	13	13.3		
F80-89 developmental disorders	2	2		
F90-98 behavioral and emotional disorders	2	2		
Unspecified diagnosis	6	6.1		
**Psychotropics**				
Antidepressant	25	26.3		
Antipsychotics	12	12.6		
Mood stabilizers	12	12.6		
Hypnotics	29	30.5		
Anxiolytics	22	23.2		
Central stimulants	5	5.3		

The suicide ideator groups with and without a suicide attempt totaled 92 participants. There were 31 suicide ideators with no suicide attempt and 61 suicide ideators with a suicide attempt.

Table 2 shows that there is a significant difference in the CWIT cognitive inhibition scores, between suicide ideators without an attempt and suicide ideators with an attempt of suicide [*t* (94) = 1.61, *p* = 0.05]. There were no other significant differences on other CWIT conditions or on the BRIEF conditions.

**Table 2:** Demographic and clinical characteristics of suicide ideators without suicide attempt and suicide ideators with suicide attempt, *N* = 92.

	Suicide ideators	Suicide attempters	*p* value
Female *n* (%)	16 (48%)	34 (44%)	0.71
Depression mean (SD)	22.8 (7.3)	25.0 (7.1)	0.19
**STROOP**			
Naming color mean (SD)	7.51 (2.9)	7.11 (3.0)	0.54
Reading color mean (SD)	8.65 (2.8)	8.38 (3.0)	0.69
Inhibition mean (SD)	9.93 (3.1)	8.53 (3.3)	0.05
Shift mean (SD)	8.13 (3.5)	8.31 (3.4)	0.80
**BRIEF**			
Global executive index	61.90 (10.2)	62.54 (10.6)	0.78
Behavior regulation index	59.68 (9.5)	60.89 (10.0)	0.58
Meta cognition index	64.70 (11.7)	63.49 (10.4)	0.62
Inhibit	58.58 (11.0)	61.16 (10.2)	0.27
Shift	60.32 (14.2)	61.30 (10.6)	0.71

Logistic regression analysis was conducted to test the predictability of cognitive control in suicide ideators and suicide attempters. Suicide ideators without suicide attempt were coded as “0” and suicide ideators with a suicide attempt were coded as “1.” For this analysis, cognitive shift and cognitive inhibition from the CWIT were selected as the main measures of cognitive control, in addition to the subindex inhibit and shift from BRIEF-A.

The Nagelkerke R Square was 0.31. This model comparing suicide attempters with suicide ideators found inhibition (*p* < 0.001) and shift (*p* < 0.001) to be contributing factors. The OR of inhibition of 0.6 showed a negative relationship to suicide attempt and the OR for shift of 1.4 showed a positive relationship to suicide attempt (Table 3).

**Table 3:** Logistic regression predicting suicide ideators vs. suicide attempter.

Variables	OR	Confidence interval	*p*-value
Gender	1.2	0.40–3.59	0.76
Depression	1.1	1.00–1.17	0.06
Inhibition STROOP	0.6	0.45–0.80	0.00
Shift STROOP	1.4	1.13–1.86	0.00
Inhibit BRIEF	1.0	0.96–1.09	0.54
Shift BRIEF	1.4	0.93–1.03	0.42

## Discussion

Suicide attempters showed poorer attentional control compared to suicide ideators, whereas attentional control combined with sifting ability contributed to the difference between the suicide ideation group and suicide attempt group. These results support the findings of previous research which indicated that suicide ideators and suicide attempters show different levels of cognitive control (Burton et al., 2011; Richard-Devantoy et al., 2014; Saffer and Klonsky, 2018).

Contrary to our prediction, the differences in cognitive control between suicide ideators and attempters did not reveal itself in the self-report This difference between subjective and objective measures could suggest that patients are not aware of their cognitive control weaknesses.

A higher cognitive shift test score and a lower attention control score suggested that a patient belonged to the suicide attempt group. What makes a suicide attempt possible could be a combination of cognitive strengths, cognitive deficits, and emotional strains. Careful interpretations of cognitive deficits concerning suicide ideation and suicide attempts are necessary to gain a better sense of which combination of cognitive deficits and cognitive strengths are significant in suicide prevention.

Poorer attention control of suicide attempters supports the findings of several meta studies (Richard-Devantoy et al., 2012). Longer response time in interference condition for suicide attempters has also been found in several studies (Keilp et al., 2013; Loyo et al., 2013). Thus, this cognitive control weakness could be understood as one of the first signs of alterations in the selective attention system (Keilp et al., 2008).

The risk of suicide attempt may be associated with poor attention control and better control in cognitive shift. More research on the combination of attention control and shift is needed to verify the strength of this cognitive pattern. Our results support a focus on specific cognitive control functions in suicide prevention work. Suicidal individuals could benefit from an attentional control level test since this could alert them to the ways in which this cognitive deficit affects their behavior. Finally, offering attentional control training could be beneficial in suicide prevention. A pilot study of high-suicide risk outpatients receiving mindfulness-based interventions, cognitive therapy, and safety planning for 9 weeks improved their attention control score in the CWIT interference condition (Chesin et al., 2016).

Some limitations of this study are the lack of information about the patients’ baseline of cognitive control and that the sample size for the regression analysis is rather small. This study has only tested two aspects of cognitive control: attention control and cognitive shift. Future studies should include a larger sample size and other aspects of cognitive control, such as planning and decision making. There is a need for more research on how cognitive control is related to suicide ideation and suicide attempt, and future studies should also include longitudinal measures. Finally, there is a need to focus on the process from suicide ideation to suicide attempt, and how different cognitive control functions and risk factors work together.

## Data Availability Statement

The raw data supporting the conclusions of this article will be made available by the authors, without undue reservation.

## Ethics Statement

The studies involving human participants were reviewed and approved by Norwegian Regional Committees for Medical and Health Research Ethics. The patients/participants provided their written informed consent to participate in this study.

## Author Contributions

SB contributed to study conception and design, project planning, acquisition of data, analysis and interpretation of data, and drafted the manuscript. NL contributed to study conception and design, project planning, and critical revision of the manuscript. VH contributed to study design, project planning, analysis and interpretation of data, and critical revision of the manuscript. All authors contributed to the article and approved the submitted version.

### Conflict of Interest

The authors declare that the research was conducted in the absence of any commercial or financial relationships that could be construed as a potential conflict of interest.
